# Structure of an Engineered β-Lactamase Maltose Binding Protein Fusion Protein: Insights into Heterotropic Allosteric Regulation

**DOI:** 10.1371/journal.pone.0039168

**Published:** 2012-06-14

**Authors:** Wei Ke, Abigail H. Laurent, Morgan D. Armstrong, Yuchao Chen, William E. Smith, Jing Liang, Chapman M. Wright, Marc Ostermeier, Focco van den Akker

**Affiliations:** 1 Departments of Biochemistry, Case Western Reserve University, Cleveland, Ohio, United States of America; 2 Department of Chemical and Biomolecular Engineering, Johns Hopkins University, Baltimore, Maryland, United States of America; University of Queensland, Australia

## Abstract

Engineering novel allostery into existing proteins is a challenging endeavor to obtain novel sensors, therapeutic proteins, or modulate metabolic and cellular processes. The RG13 protein achieves such allostery by inserting a circularly permuted TEM-1 β-lactamase gene into the maltose binding protein (MBP). RG13 is positively regulated by maltose yet is, serendipitously, inhibited by Zn^2+^ at low µM concentration. To probe the structure and allostery of RG13, we crystallized RG13 in the presence of mM Zn^2+^ concentration and determined its structure. The structure reveals that the MBP and TEM-1 domains are in close proximity connected via two linkers and a zinc ion bridging both domains. By bridging both TEM-1 and MBP, Zn^2+^ acts to “twist tie” the linkers thereby partially dislodging a linker between the two domains from its original catalytically productive position in TEM-1. This linker 1 contains residues normally part of the TEM-1 active site including the critical β3 and β4 strands important for activity. Mutagenesis of residues comprising the crystallographically observed Zn^2+^ site only slightly affected Zn^2+^ inhibition 2- to 4-fold. Combined with previous mutagenesis results we therefore hypothesize the presence of two or more inter-domain mutually exclusive inhibitory Zn^2+^ sites. Mutagenesis and molecular modeling of an intact TEM-1 domain near MBP within the RG13 framework indicated a close surface proximity of the two domains with maltose switching being critically dependent on MBP linker anchoring residues and linker length. Structural analysis indicated that the linker attachment sites on MBP are at a site that, upon maltose binding, harbors both the largest local Cα distance changes and displays surface curvature changes, from concave to relatively flat becoming thus less sterically intrusive. Maltose activation and zinc inhibition of RG13 are hypothesized to have opposite effects on productive relaxation of the TEM-1 β3 linker region via steric and/or linker juxtapositioning mechanisms.

## Introduction

Allosteric regulation is a common mechanism cells utilize to regulate protein activity. Heterotropic allosteric proteins have a ligand binding site distant from the substrate binding site to allow conformational changes to be transmitted from the ligand regulatory binding site. As such, engineered proteins with allosteric properties have attractive applications including molecular biosensors, therapeutic proteins, and gene regulation [1], [2]. One approach to engineering allosteric proteins is to mimic non-homologous recombination via domain/module combinations and rearrangements. This approach can develop complex new functions involving molecular recognition and regulation [3] and can lead to development of analytical molecular sensors [4]. A successful example of this approach is the allosteric protein RG13 which is a molecular switch created by recombining the nonhomologous genes encoding MBP and TEM-1 β-lactamase and subjecting the resulting library to evolutionary pressure [5]. In brief, a circular permutated TEM-1 gene was randomly inserted into the gene encoding MBP. The constructs were subjected to an *in vivo* selection for high ampicillin hydrolysis activity in the presence of maltose, followed by an *in vitro* screen for variants with maltose-dependent rate of β-lactam hydrolysis. The resulting RG13 construct was demonstrated, in the presence of maltose, to have β-lactamase activity comparable to the wild-type TEM-1 β-lactamase but RG13’s activity (*k*_cat_/*K*_m_) decreased 25-fold in the absence of maltose [5]. As a consequence, the *RG13* gene confers a maltose-dependent ampicillin resistance to *E. coli* cells [5]. Intriguingly, RG13 activity was serendipitously found to be negatively regulated by Zn^2+^ at low µM concentrations in a non-competitive and reversible mode, a characteristic that neither parent protein MBP nor TEM-1 possesses [6].

**Figure 1:**
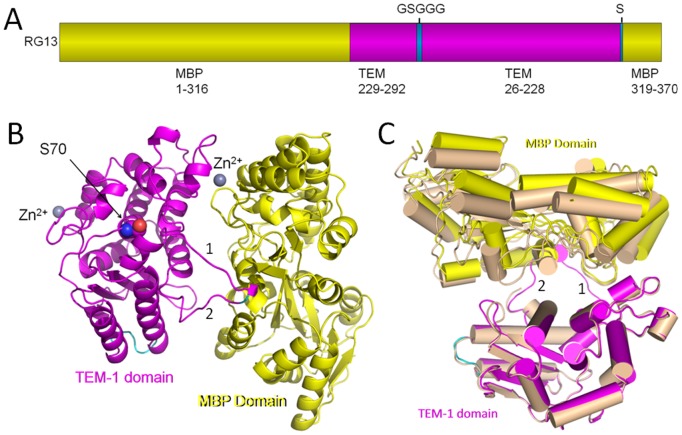
Overall structure of RG13. (A) RG13 primary structure. (B) Schematic diagram of RG13 crystal structure with zinc ions bound. TEM-1 and MBP domains are colored magenta and yellow, respectively. Zinc ions are shown as grey spheres and the engineered GSGGG linker is colored cyan. To indicate the position of the TEM-1 active, its catalytic serine residue is highlighted in spheres. The linker 1 and linker 2 regions between the MBP and TEM-1 subdomains are labeled as ‘1′ and ‘2′, respectively; (C) Superposition of the two RG13 crystal forms of the TEM-1 domain reveals a slightly different orientation of the MBP domain after TEM-1 domain superpositioning.

The primary structure of RG13 is unusual in that it is a 72kD fusion protein of TEM-1 inserted into MBP [5]. Due to the circularly permutated TEM-1 gene, the new TEM-1 N-terminal residue 229 is inserted after MBP residue 316 whereas the new C-terminal residue of TEM-1, residue 228, is connected to MBP residue 319 via an extra serine residue [5]. The original N- and C-terminal residues of TEM-1 are connected via a linker GSGGG. In total, there are two connections between the TEM-1 and MBP domains in RG13 (Figure 1A).

To our knowledge, the structural basis for how allostery is achieved by non-homologous domain insertion has not been described. Initial insights into the allosteric mechanism of RG13 were obtained by the Ostermeier lab with mutational studies indicating that the extent of the domain closure angle in the MBP domain, which is maximally achieved by maltose binding, is responsible for affecting TEM-1 activity in RG13 [5], [7]. A recent NMR study compared the NMR data of RG13 in the absence/presence of maltose with data from previous NMR studies of MBP and TEM-1 and provided evidence that: (1) the individual MBP and TEM-1 domain structures in RG13 are substantially conserved; (2) the TEM-1 active site of RG13 is relatively unperturbed in the presence of maltose; and (3) in the absence of maltose the active site of RG13 is slightly perturbed with possible displacement of several residues [8]. Less is known about the negative allosteric regulation of RG13 by Zn^2+^. Previous mutagenesis studies involving single and double mutations have potentially ruled out all Cys and His residues for Zn^2+^ binding [6] but did suggest a possible role for TEM-1 residues H26 and H289 in the zinc inhibition mechanism. Upon mutating both of these residues to alanine, the Zn^2+^ *K_i_* increased 30-fold although a corresponding loss of Zn^2+^ affinity was not observed [6].

We are interested in further delineating the mechanism of switching of RG13 as to how the TEM-1 activity is positively regulated by the allosteric effector maltose and negatively regulated by Zn^2+^. The RG13 crystal structure with mM Zn^2+^ concentration in the crystal presented here shows RG13 with a distorted TEM-1 domain with a zinc ion bound at an inter-domain site. The structure shows that this zinc ion bridges the TEM-1 and MBP at an additional contact point away from the linker region between the two domains thereby negatively affecting the conformation of this linker region. An analogous distortion of this linker may be key for RG13’s compromised catalytic activity in the absence of maltose. To gain additional insights, we carried out mutagenesis studies to probe both the linker region and the zinc site as well as carried out molecular modeling to investigate RG13 with an intact TEM-1 domain.

## Results

### Overall Structure

Two types of crystals of RG13 were grown in the presence of high zinc concentration (2.5 mM). These crystals belonged to space groups P1 and C2 and were refined to 2.30 and 2.29Å resolution, respectively (Table 1). The structure reveals that despite the domain shuffling, circular permutation, and different connections, RG13 contains two folded domains comprised of the regulatory MBP and the catalytic TEM-1 (Figure 1B). The overall dimensions of each RG13 molecule are roughly 50×70×70Å with the TEM-1 and MBP domains lying alongside each other connected by two linkers. The two RG13 molecules in the P1 space group have a similar conformation as the root-mean-square-deviation (r.m.s.d.) for Cα atoms is 0.39Å (for 636 residues). Superpositioning of the RG13 molecule from the C2 space group onto each of two molecules in the P1 space group yields r.m.s.d.’s of 1.7 Å (619 residues) and 1.6 Å (615 residues) for molecules A and B, respectively. The electron density for most of RG13 is well resolved including most, but not all, of the TEM-1 active site (Figure S1). The two connecting loops between TEM-1 and MBP could only be resolved in the P1 space group (in both monomers) (Figure S1) although these loops, not unexpectedly, have higher temperature factors than most of the rest of the protein (Figure S1); part of these linker regions comprising residues 317–327 and 583–584 are not included in the C2 space group structure due to lack of electron density. Similarly, the GSGGG linker introduced to circularly permute TEM-1 is resolved in the electron density maps but also has somewhat higher temperature factors compared to the cores of the two domains (Figure S1).

**Table 1:** Data collection and refinement statistics.

Data collection			
	space group	*P1*	*C2*
	cell dimensionsa, b, c (Å), α, β, γ (deg)	48.32, 74.18, 103.13, 83.54, 77.64, 89.98	124.12, 47.78, 107.87, 90.00, 114.17, 90.00
	wavelength (Å)	1.0810	1.0810
	resolution (Å) a	50.00–2.30 (2.38–2.30)	50.00–2.30 (2.38–2.30)
	Rsym	5.4 (22.3)	5.1 (32.8)
	I/*σ*I	12.6 (3.8)	15.4 (2.7)
	Completeness (%)	95.9 (95.5)	88.0 (88.2)
	Redundancy	1.9 (1.9)	2.3 (2.3)
**Refinement**			
	Resolution range (Å)	31.45–2.29 (2.294–2.354)	42.32–2.30 (2.360–2.301)
	no. of reflections	56130	22012
	Rwork/Rfree	23.2/29.2	23.3/29.1
	no. of atoms: protein, zinc ion, water	9851, 4, 490	4830, 2, 134
	rmsd b		
	bond length (Å)	0.007	0.006
	bond angles (deg)	0.960	0.942
	average B-factors (Å2)		
	Protein	34.385	41.047
	zinc ion	31.573	40.825
	Water	34.750	36.184
	Ramanchandran plot statistics (%)		
	core regions	91.7	92.1
	additional allowed regions	8.1	7.5
	generously allowed regions	0.2	0.2
	disallowed regions	0.0	0.2

a Numbers in parentheses refer to the highest resolution shell.

b rmsd, root-mean-square deviation.

The orientation between RG13’s TEM-1 and MBP domains in the C2 space group is somewhat different compared to that in the P1 space group (Figure 1C). When superimposing the TEM-1 domains of the RG13 structures, the MBP domain orientation between the two different space group structures is different by about 8.5°: the MBP domain appears to pivot across the TEM-1 surface with MBP residues furthest from the TEM-1 domain shifted more compared to the MBP residues closest to the TEM-1 domain likely a consequence of the bridging Zn^2+^ and linkers restrictions. These observed inter-domain orientational differences indicate a degree of flexibility in orientation between the two domains, not unexpectedly due to the presence of the somewhat more mobile linker regions and the limited contacts between the two domains. The additional Zn^2+^ ion contact between MBP and TEM-1 will be discussed next.

### Zn^2+^ Binding

In addition to the two RG13 monomers in the P1 space group, there are four zinc ions present with two zinc ions bound per monomer. Similarly, there are two zinc ions present in the C2 space group structure of RG13. The zinc ions are readily identifiable being the strongest difference Fourier density features in difference density maps (Figure S2). The Zn^2+^ ions bound to the three different RG13 monomers can be grouped in two different classes: the main Zn^2+^ ion, which bridges the TEM-1 and MBP domains, and the non-interdomain bridging Zn^2+^ ion. The main Zn^2+^ ion in the C2 RG13 structure bridges the TEM-1 and MBP domains via residues H468 (*H112,* TEM-1 residue numbering shown in italics and brackets from here on forward), residue E477 (*E121*), and both oxygens of residue D164 (MBP residue) (Figure S2). In the P1 structure, residue E477 (*E121*) has slightly shifted away and therefore the main Zn^2+^ ion only interacts with H468 (*H112*) and both oxygens of D164 (Figure S2). The main Zn^2+^ ion binding site in RG13 is distant from the active site being 20Å from the Oγ atom of the catalytic S426 (*S70*) residue. The (other) non inter-domain bridging Zn^2+^ ion makes liganding interactions with residues H509 (*H153*) and H514 (*H158*) of the TEM-1 domain (in C2 space group, Figure S2). A similar zinc binding site is also present in the P1 space group involving the same H509 and H514 residues but this zinc binding site is expanded and also includes ligands from a crystallographically related RG13 MBP domain: H39′ and Y17′ (Figure S2).

### MBP Domain

**Figure 2:**
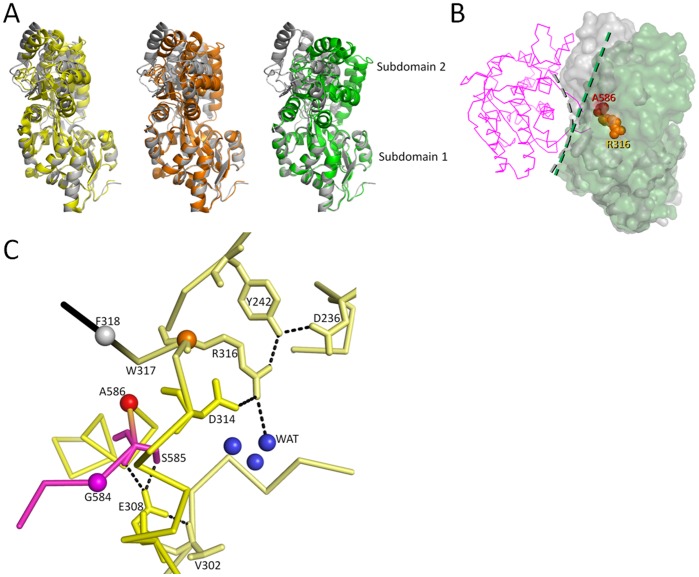
Structural analysis of the MBP subdomain angle variations. (A) Superposition of subdomain 1 of MBP reveals different domain closure angles of subdomain 2. The superposed structures include uncomplexed MBP structure (PDB ID: 1OMP, grey), RG13 structure of P1 space group (yellow), RG13 structure of C2 space group (wheat), and maltotriose complexed MBP structure (PDB ID: 3MBP, green). Subdomain 1 of MBP with RG13 is defined by residues 1–109 and 261–316 and subdomain 2 is defined as 110–260 & 586–637. (B) Hypothesized surface change of MBP upon maltose binding in the vicinity the fusion sites R316 and A586 (A319). The surface of the maltose-bound MBP (green) and uncomplexed MBP domains (grey) are shown within the RG13 framework with TEM-1 depicted in a magenta Cα trace. The surface change from concave (grey curved interrupted line) to flat (green straight interrupted line) going from uncomplexed to maltose-bound is indicated. (C) Close-up view of the fusion site between MBP and TEM-1 showing the RG13 linker anchor residues R316 and S585 and their interactions with the rest of the MBP domain.

MBP adopts a periplasmic binding fold and is capable of undergoing a ligand-induced subdomain closure in which the ligand maltose binds to both subdomain halves of MBP leading to a domain closure of ∼35° [9]. Superpositioning shows that the MBP domain of RG13 conformation is more similar to the uncomplexed MBP structure (PDB ID: 1OMP). The latter superpositioning yielded relative low r.m.s.d. values of 1.25Å monomer A in P1, 1.39Å for monomer B in P1, and 1.81Å in space group C2 compared to superpositioning with the maltotriose complexed MBP structure (PDB ID: 3MBP) which yielded larger r.m.s.d.s. of 2.76 (P1 monA), 2.80 (P1 monB), and 2.0 Å (C2) for about 360 Cα atoms (Figure 2). This result is in agreement with a subdomain rotation analysis indicating that RG13 is in a more unliganded open conformation in both P1 and C2 spacegroups since its domain closure angle is about 10° and 17°, respectively, compared to that of the uncomplexed MBP structure (Figure 2). The linker regions, where TEM-1 and MBP are fused, extend from the backside of the MBP domain opposite of where maltose binds. Therefore, the TEM-1 domain in RG13 will see a changing surface upon maltose binding going from concave to more flat (Figure 2B). This will have likely steric consequences for the linker tension between the MBP and TEM-1 domains as will be discussed later. The residues of the linkers closest to the MBP domain that appear to be anchoring the linker to MBP are residues S585 and R316 as the mainchain and sidechain residues of these residues make numerous stabilizing interactions in the P1 space group (Figure 2C). Furthermore, S585 and R316 are also the last residues visible in the C2 RG13 structure whereas the rest of the linker regions are too disordered to be modeled in this C2 spacegroup.

### TEM-1 Domain

**Figure 3:**
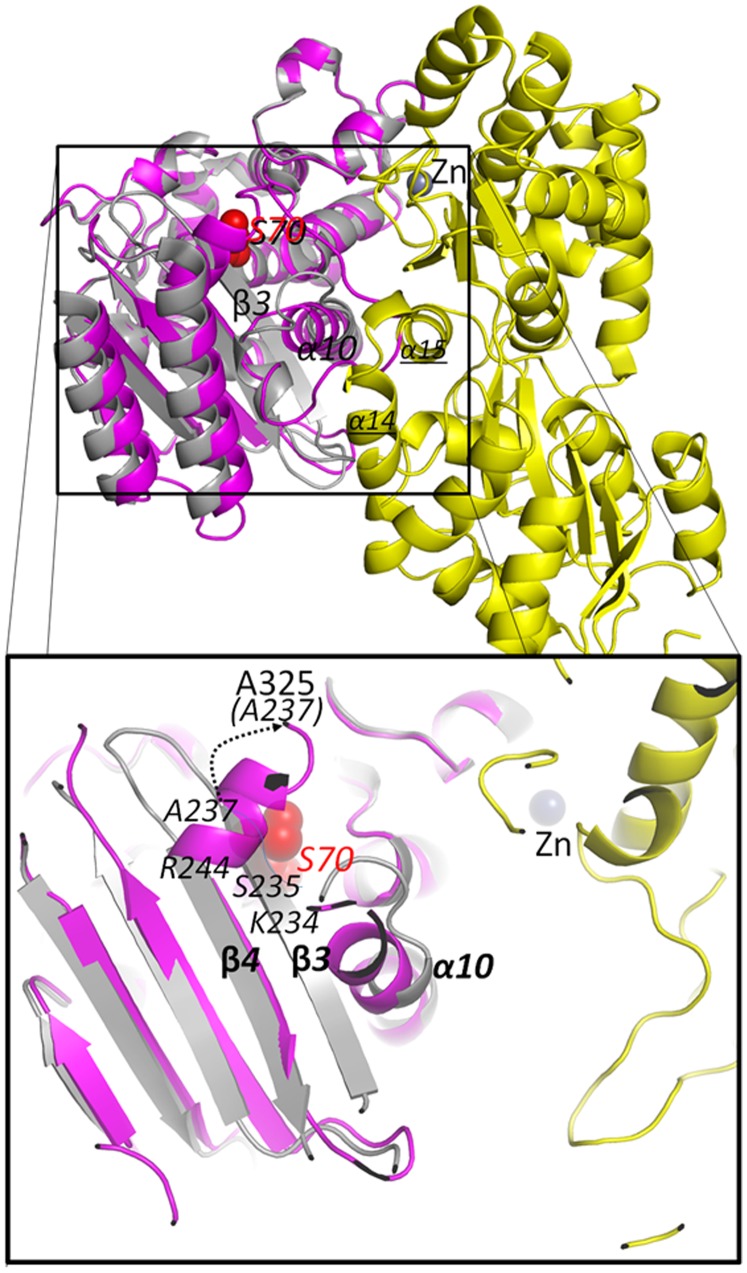
Active site changes in TEM-1 domain of RG13 compared to wt TEM-1 structure. Superpositioning of wt TEM-1 structure onto the TEM domain in RG13 reveals displacement of β3 strand and part of β4 strand (inset is a zoomed in view with secondary structure elements in transparent representation). The TEM-1 β4 strand is mostly intact yet starts diverging at position R244. The α10 TEM-1 helix which is observed to be more extended in RG13. Also labeled and underlined are the MBP helices α14 and α15 that form part of the linker anchor points in the RG13 fusion protein. TEM-1 β3 strand residue positions K234, S235, A237, and β4 strand residue K244 are indicated as well as the new position of A237 with the RG13 sequence A325. The zinc ion is indicated via a grey sphere.

TEM-1 is a β-lactamase that hydrolyses bicyclic β-lactam compounds that contain a carboxyl moiety and a carbonyl moiety. Key active site elements for TEM-1 are the catalytic S70, the carboxyl binding pocket, the oxy-anion hole for carbonyl oxygen, and the deacylation-water priming E166 residue. The TEM-1 domain of RG13 has an overall fold similar to the wild-type TEM-1 structure (PDB ID: 1ZG4) with r.m.s.d. values of 1.02Å (P1 molA), 1.02 Å (P1 molB), and 1.1 Å (C2) for ∼240 Cα as calculated using COOT [10]. The most significant structural change in the TEM-1 domain of RG13, compared to the wt TEM-1 structure, is the displacement of TEM-1 residues 229–244 which comprises the critical active site β3 strand, part of the β4 strand, and the connecting loop between these strands. This TEM-1 active site section in RG13 is now part of the linker 1 connecting the MBP and TEM domains (Figures 1B and 3). In addition, the preceding residues 214–228 are in a somewhat different conformation as the TEM-1 α10 helix extends in RG13 forming a longer helix (Figure 3). Despite these substantial active site differences of missing active site β-strands, the positions of most other active site TEM-1 residues including S70, Y105, S130, N132, E166, and N170 (in TEM-1 residue numbering) are relatively unchanged in RG13. The β3-strand plays an important role for TEM-1 activity because it forms one of the active site walls providing part of both the carboxyl binding pocket (via S235) and oxy-anion hole for the carbonyl oxygen (via the backbone nitrogen of residue A237) which are thus no longer in the correct position in the Zn^2+^ bound structure of RG13. β3-strand residue K234 is also critical for β-lactamase functioning [11] and is no longer in the native TEM-1 position (Figure 3). Furthermore, β4-strand residue R244, which is also involved in electrostatic interactions with the β-lactam carboxyl moiety [12], has also shifted. This β3-β4-strand section, comprising TEM-1 residues 229–244 is in close proximity to one of the fusion sites, i.e. residue 229, between TEM-1 and MBP (Figures 1 and 3) [5]. In addition to lacking some key active site features, the entry to the TEM-1 active site in RG13 is partially blocked by this displaced region that now forms a short helix (Figure 3). Taken together, loss of β3-β4 strand section adopting a new conformation, now sterically blocking the active site, likely explains the lack of activity of the Zn^2+^-bound RG13 structure at mM concentration of Zn^2+^. Further away from the active site, residues V216 and A217 have also shifted somewhat and these shifts could also affect enzyme activity as those residues have been shown to not tolerate changes [13]. However, this disturbance is likely not a main contributor to the loss of activity.

### Mutagenesis

To probe the importance of the crystallographically observed main Zn^2+^ binding site (Figure S2) for Zn^2+^ inhibition of RG13, we mutated the liganding residues D164, H468, and E477 to alanine. In addition to the single mutants D164A and E477A, we also constructed the D164A/H468A double mutant as well as the D164A/H468A/E477A triple mutant. All proteins were purified and confirmed to maintain their ability to be activated by maltose. Both single mutants D164A and E477A were inhibited by Zn^2+^ to the same extent as wild-type RG13. The *K*_i_ for the D164A/H468A double mutant was about 4-fold higher; however, the triple mutant exhibited a *K_i_* that was only 2-fold higher compared to RG13’s *K*_i_ being (Table 2). The inhibition of all mutants fit well to a model of a single Zn^2+^-binding site. This data indicate that the D164A/H468A/E477A site is not singly responsible for the µM Zn^2+^ inhibition although some modest contribution to Zn^2+^ inhibition was observed.

**Table 2:** Kinetic constants for nitrocefin hydrolysis and Zn^2+^ inhibition of TEM-1, RG13, and mutants thereof.

Protein	*k*_cat_ (s^−1^)	*K*_m_ (µM)	*k*_cat_/*K*_m_ (s^−1^µM^−1^)	*K*_i_ for Zn^2+^ (µM)	Ref.
TEM-1	900	110	8.18	340–1100	[5], [6]
RG13	200±40	550±120	0.36	2.7±0.1, 3.2±0.5	[5], [6], this study
RG13+maltose	620±60	68±4	9.12	2.1±0.2	[5], [6]
RG13 D164A/H468A	nd*	Nd	nd	8.7±1.4	this study
RG13 D164A/H468A/E477A	nd	Nd	nd	4.1±1.0	this study
RG13 H375A/H382A	nd	Nd	nd	73±5	[6]

* nd, not determined; however, it was confirmed that all mutants were activated by maltose like RG13 using nitrocefin assays (100 µM nitrocefin) in the presence and absence of maltose.

**Figure 4:**
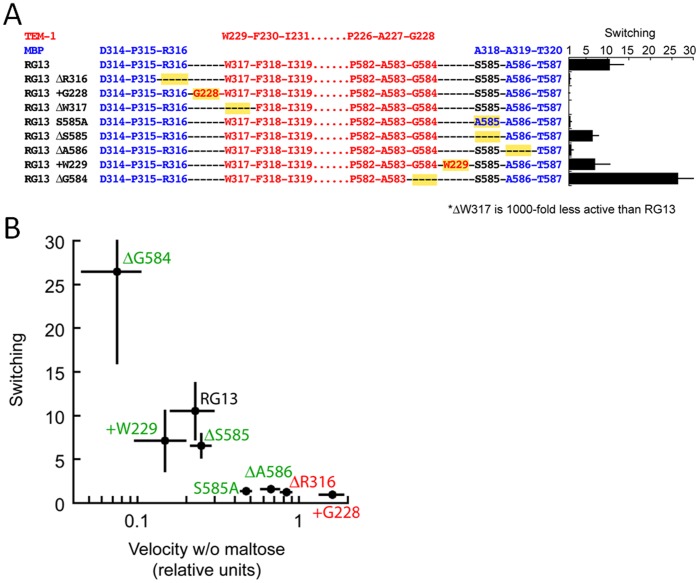
The effect of mutations in linker 1 and linker 2 on maltose-induced switching. (A) Mutation locations and their effect on switching. Residues derived from MBP are colored blue and those from TEM-1 are colored red. The corresponding numbers in the wt proteins are indicated above. Mutations relative to RG13 are highlighted in yellow. The switching activity (fold increase in catalytic activity in the presence of a saturating concentration of maltose) of each mutant is indicated on the right. Error bars are standard deviations (n≥3). (B) Switching activity shown as a function of enzyme activity in the absence of maltose. Mutations to linker 1 are shown in green and mutations to linker 2 are shown in red.

To probe the importance of the linker 1 and linker 2 regions at the site of fusion between TEM-1 and MBP, a set of point mutations and single amino acid additions/deletions were constructed (Figure 4A). Switching was measured as a ratio of initial rates +/− maltose at a particular concentration of substrate that is around the K_m_ (hence RG13 switching is not 25-fold). Mutations to the linker 1 region either greatly diminished activity (e.g. ΔW317) or resulted in a loss of switching. The effects of mutations to the linker 2 varied from a loss of switching to a 2-fold increase in switching (e.g. ΔG584). As a general rule, mutations that caused an increased enzyme activity in the absence of maltose also decreased switching (Figure 4B). Similarly, the ΔG584 mutation increased switching by decreasing the activity in the absence of maltose. To test specifically potential MBP anchor residues S585 and R316 (Figure 2B–2C) we mutated residues S585 to an Ala (S585A) and separately deleted residue R316 (ΔR316); these mutants were found to have abrogated maltose-switching. Overall, the linker 1 region could not tolerate any of the three changes made to the linker without losing switching or overall activity whereas 3 out of 5 linker 2 modifications still yielded substantial switching activity. These results point to a critical role for linker 1 in maltose switching likely due to being adjacent to the key TEM-1 active site β3 strand section. In accord with linker 1 and its anchoring residue(s) being critical in maltose switching, loss of switching and restoration of maximal wt activity in the absence of maltose can be achieved by increasing slack immediately adjacent to the linker 1 anchor residue R316 via insertion of a Gly residue (+G228) (Figure 4). Linker 2 is important as well yet positive and negative effects on switching can be observed upon altering the linker. As mentioned above, mutating the anchor residue S585 to an Ala caused loss of switching. Deleting either residue adjacent to S585 (i.e. G584 or A586) had completely opposite effects on switching and points to an important role for A586. Keeping the entire α15 anchoring region intact (i.e. residues S585-A586) yet deleting G584 yielded a hyper maltose switching variant (ΔG584). In contrast, deleting the A586 residue, which makes key hydrophobic interactions and is part of the α15 helix of MBP, eliminates the switching. This is likely due to disruption of this S585-A586 α15 helix anchor region as deletion of A586 will shift S585 to the position of this deleted residue but this residue is likely unable to be accommodated due to the serine’s larger size and more hydrophilic nature compared to alanine. The significant switching activity still present for the variant with the deletion of residue S585 is likely a consequence of two opposite effects negating each other. The negative effect of removing the anchoring abilities of residue S585, as observed for the S585A mutation, is compensated for by the hyper-switching effect of shortening the linker 2 region by the deletion as observed for the ΔG584 mutant (while maintaining at least the A586 part of the α15 helix anchor region). The insertion of a tryptophan in the linker 2 region (+W229) still maintained some switching activity but the structural consequences of increasing slack via insertion of the largest hydrophobic amino acid are not easily explained since this residue might generate additional (hydrophobic) anchoring interactions.

### Molecular Modeling of RG13

**Figure 5:**
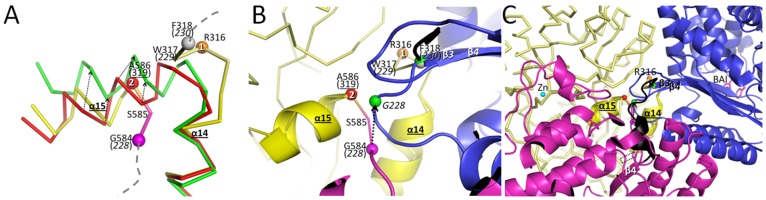
Modeling of RG13 fusion protein with a catalytically competent TEM-1 domain near the MBP fusion site to relay maltose-induced conformational changes. (A) Superpositioning of the subdomain 1 of MBP (residues 1–109 and 261–316) from maltose-free MBP (red, PDBid 1OMP), maltotriose-bound MBP (green, PDBid 3MBP), and RG13 P1 space group structure (yellow). MBP anchor/fusion points labeled ‘1′ (R316) and ‘2′ (A586(*319*)) to which TEM-1 is fused are labeled and colored as orange and red spheres, respectively. The respective MBP helices to which these two MBP anchor points are adjacent to are helices α14 and α15, respecively. The positions of S585, introduced as part of constructing RG13 (Figure 1A), and the TEM-1 residues 228, 229, and 230 in the RG13 fusion protein are labeled; the other connecting TEM-1 residues are shown as dashed grey lines. Shifts in the Cα positions of the α15 helix going from unbound to maltotriose bound MBP conformation are indicated by dashed arrows. (B) Close-up view of RG13 fusion site showing the MBP (yellow) and TEM-1 domains (magenta) of RG13, and the modeled position of an intact TEM-1 domain (blue, PDBid 1ERO which includes a boronic acid inhibitor to indicate the position of the active site). Modeling target points F318 (*230)* and G584 (*228*) in RG13 are shown as a grey and magenta sphere, respectively, and the equivalent positions F230 and G228 in wt TEM-1 are depicted as green spheres. The movement that is needed for G584 (*228*) in RG13 to reach the G228 position as found in wt TEM-1 is depicted by a dashed arrow. The MBP anchor point helices α14 and α15, are shown in yellow helix cartoon representation. TEM-1 β3 and β4 strands are labeled; (C) zoomed out view as in (B). TEM-1 bound boronic acid inhibitor (BAI) is shown in magenta stick representation to pinpoint the position of the TEM-1 active site. The black line and small black helix depict the RG13 residues that normally form the TEM-1 β3 and part of β4 strand yet now form the linker between the TEM-1 and MBP domains in the zinc inhibited RG13 structure. The rectangular dashed box in RG13 highlights the position of where the β3 strand used to be in an intact TEM-1 conformation.

To gain insights into how an intact TEM-1 structure would fit within the RG13 framework, we employed a rigid body modeling approach. In short, this was done by modeling an intact TEM-1 molecule (with the β3-β4-strand section in the wild-type position) in close proximity to the maltose-bound MBP within the RG13 framework (Figure 5). Firstly, maltotriose bound (PDB id: 3MBP) and unbound (PDB id: 1OMP) MBP structures were superposed onto the RG13-Zn^2+^ crystal structure by superpositioning of residues 1–109 and 261–316 which is one of the sub-domains of MBP. The superposition indicated that the MBP anchor point helices α14 and α15 move relative to each other upon maltose/maltotriose binding (Figure 5A). Subsequently, G584 (*G228*) and F318 (*F230*) were chosen as the modeling target points to attach TEM-1 to the MBP in RG13 thereby keeping the R316/W317 and S585 MBP anchor regions intact (we kept W229 in its RG13 position)(Figure 5B). In the modeled TEM-MBP RG13-like complex, the TEM-1 domain has a three dimensional location fitting reasonably well only some van der Waals clashes as the two domains are in very close proximity (Figure 5C). Nevertheless, in the modeled complex, the TEM-1 G228 modeling target point is in closer proximity to F318 compared to where it is in the RG13-Zn structure, being RG13 residue G584 (Figure 5B). As noted above, this target attachment point G584 is however anticipated to shift in closer proximity upon maltose binding based upon superpositioning of the maltose-free and maltose/maltotriose-bound MBP (Figure 5A). Thus, the movement extrapolated to occur upon maltose binding (Figure 5A) and the movement needed to occur to have residues G584 (*228*) and F318 (*230*) in wt TEM-1 proximity (Figure 5B) are in roughly the same direction suggesting that maltose binding could juxtaposition TEM-1 residues G228 and F230 to a shorter more native like distance. This shift is likely key as residue G228 is the start of the important active site β3 strand. Therefore, we propose that maltose binding likely tweaks this RG13 region such that the β3-β4 strand region involving TEM-1 residues 228–244 is relaxed enough to fit correctly and productively in the active site such that RG13 yields wild-type TEM-1 activity in the presence of maltose. Concomitantly, the local surface change from concave to flat near the linker attachments points on MBP upon maltose binding (Figure 2B) will likely result in less steric clashes between the two domains and also less pull on the TEM-1 β3 strand.

## Discussion

The crystal structure of RG13 bound to the non-competitive Zn^2+^ inhibitor provides insights into the heterotropic allosteric signaling of RG13 although the relative importance of the inter-domain Zn^2+^ site is not immediately evident. The structure reveals that the TEM-1 and MBP domains are in close proximity to each other connected via two linkers and a bridging Zn^2+^ ion (Figure 1B). Whereas the MBP domain is quite intact adopting a relatively open/unliganded conformation, the TEM-1 domain active site is compromised with the active site β3-β4 strand section mostly displaced from its wild-type TEM-1 position with this displaced section now partially blocking substrate entry while also forming a linker between the two domains (Figure 3). Comparison of two different space group RG13 structures reveals that there is still some flexibility in the orientation between the MBP and TEM-1 domains as well as within the two lobes of MBP (Figure 1C and 2A). The zinc ion at high mM concentration therefore seems to inhibit RG13 by generating an additional anchor point between the two domains and therefore acts to ‘twist-tie’ the original anchor points and linkers such that these linkers are contorted and no longer have enough slack to be inserted productively into the active site (in particular β3-β4 strand residues 226–244). The β-strand expulsion and re-insertion is reminiscent of how the bloodclotting serpin anti-thrombin is regulated [14], [15]. The zinc ion observed at the inter-domain site acts thus as a non-competitive inhibitor binding distant from the active site yet clearly affecting β-lactamase activity. Before discussing the relative importance of the Zn^2+^ site, we will first describe the structural consistencies of the RG13 structure with the currently available data.

### Structural Agreement with Non-Crystallographic Data

**Figure 6:**
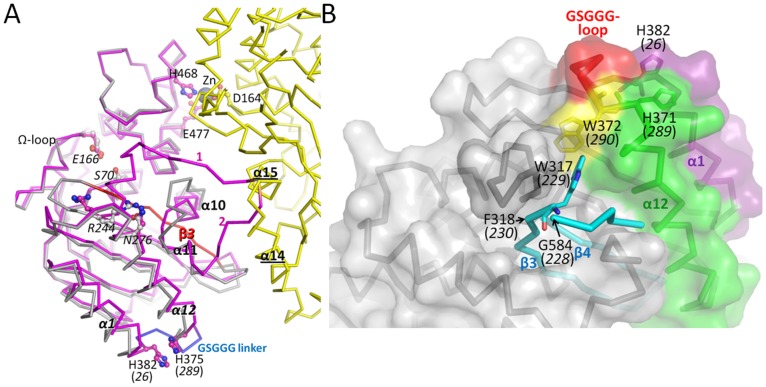
Zinc ion regulation in RG13. (A) RG13 (yellow and magenta) is superimposed onto wt TEM-1 (in grey). The crystallographically observed zinc binding ligands D164, H468, and E477 in RG13 are shown in ball-and-stick. Active site residues S70, E166, R244, and N276 are also shown. MBP is depicted in yellow with its anchor/fusion point helices α14 and α15 shown as yellow transparent rods. TEM-1 is shown in magenta with its original N- and C-terminal helices *α1* and *α12* shown in magenta transparent rods. The engineered GSGGG linker between these helices is shown in blue. The position of the original TEM-1 β3 strand position is indicated by the transparent red strand. The linkers 1 and 2 between MBP and TEM-1 are labeled 1 and 2, respectively. The positions of the RG13 residues previously shown to be involved in the zinc-mediated inhibition of RG13, H375 and H382 (i.e. TEM-1 residues 26 and 289), are shown in ball and stick and labeled. TEM-1 helix α10 is also labeled due to its close proximity to the active site, close proximity to TEM-1 α12, and observed structural differences between RG13 and TEM-1. (B) Surface representation depicting the TEM-1 fusion site and GSGGG-loop region of a modeled TEM-1 with an intact β3-β4 strand section and the GSGGG-loop within the RG13 framework. Depicted are residues H26 and H289 from the original N- and C-terminal helices of TEM-1, respectively. Also shown are residues W290 and W229 as well as the residues 230 and 228. This surface is facing the MBP domain in RG13 revealing the potential steric and structural consequences of mutations in the histidine residues.

Many crystallographically observed details of the RG13 structure correlate well with previously published data on RG13 (in the presence or absence of maltose) or are in agreement with mutagenesis data presented herein: (1) the position of the small TEM-1 α11 helix comprising residues 220–225 is perturbed compared to wt TEM-1 (Figure 6) in agreement with NMR data that pointed to a different position for this region in both the absence and presence of maltose [8]. This is not too surprising since MBP is fused to TEM-1 at nearby position 228. (2) The original C-terminal helix of TEM-1, α12, is somewhat shifted (Figure 6) as was also observed by NMR [8]. A possible explanation for this α12 shift is the shift of the flanking α11 helix and possibly the presence of the new GSGGG linker. (3) The RG13 structure contains an inhibitory inter-domain Zn^2+^ site that forces RG13 to adopt a conformation that provides a β3-strand involving mechanism regarding explaining the non-competitive inhibitory properties of Zn^2+^ [6], although the observed Zn^2+^ site is likely not singly responsible for the entire inhibitory effect (as will be discussed below). (4) MBP is observed in a mostly open/unliganded conformation since no maltose was included during the crystallization. (5) Most, but not all, of the TEM-1 active site residues are in unperturbed positions as also observed using NMR [8]. (6) The structure pointed to important structural roles for the linker anchor residues S585 and R164 as also determined by mutagenesis. (7) The N-terminal section of the TEM-1 β3 strand region adjacent to the 229 fusion site was previously pinpointed by NMR as potentially mechanistically important; this section was observed by NMR to be displaced in the absence of maltose [8]. The RG13 structure shows also a displacement of this section, albeit more drastic as the entire β3 strand is displaced. Nevertheless, both independent observations are consistent and point to a crucial role for part or the whole β3 strand which is adjacent to the 229 fusion site.

### Insights into a Maltose-Activated RG13 Structure

From the above analyses it is evident that RG13 if it has Zn^2+^ bound as observed in the crystal is in an inhibited state due to the displacement of the critical β3-β4-strand region now partially blocking the active site. There are several lines of data that indicate that in the absence of Zn^2+^, RG13 adopts a much more catalytically conducive conformation compared to the observed Zn^2+^ bound structure, even before maltose binding. First, previous kinetic studies showed that in the presence of maltose, RG13 has wt TEM-1 activity. Even when maltose is absent, there is still significant activity present since the *k_cat_*/*K*_m_ is ∼25-fold lower activity compared to wild-type TEM-1 with *k*_cat_ and *K*_m_ each only improving roughly 3- and 8-fold, respectively, upon maltose binding (Table 2). Note that if the β3-strand would be displaced entirely in the presence (or absence) of maltose, the effect on activity would be drastic as there would be no oxyanion hole, no K234 and R244, and such an active site configuration would likely therefore also have little or no catalytic activity. For example, the mutation K234T in TEM-1 affects substrate affinity 50-fold [11], and the K234A mutation in a related β-lactamase decreased *k_cat_*/K_m_ multiple orders of magnitude [16]. Second, in agreement that a Zn^2+^-free and maltose-bound RG13 structure likely adopts a more catalytically proficient conformation is that a previous NMR study found that, in the presence of maltose, the entire β3-strand can be assigned to a wt position (even in the absence of maltose, roughly two-thirds of the C-terminal part of the TEM-1 β3 strand in RG13 can be assigned to a relatively wt conformation including K234) [8]. In conclusion, it seems therefore likely that in maltose-bound RG13, the entire β3-strand is in the wt TEM-1 position. With the RG13 fusion sites between TEM-1 and MBP being at the beginning of this β3 strand (i.e. residues 228 and 229), and that this entire β3 strands adopts a wt-like position in RG13 in the presence of maltose, according to the NMR study, yielded a starting direction to model RG13 in a more activated state. This was done via rigid body modeling an active wt TEM-1 structure within an RG13 framework dictated by the fusion points (Figure 5). The rigid body modeling indicated that juxtapositioning an intact TEM-1 molecule adjacent to where it is fused to MBP in RG13 results in a very close distance between the two domains. Furthermore, the fusion point distance between TEM-1 is only moderately too large when MBP is in the unliganded conformation (Figure 5). However, this potential fusion distance mismatch is likely shortened upon the extrapolated maltose-induced MBP conformational changes (Figure 5A). This suggests that maltose brings about a conformational change such that RG13 residues G584 (*G228*) and F318(*F230*) can adopt a distance more closely observed in wt TEM-1 for these corresponding residues G228 and F230 thereby likely allowing a catalytic proficient conformation for this entire region encompassing strands β3 and β4. In addition to the fusion points juxtapositioning upon maltose binding, the MBP surface near the fusion sites becomes less sterically repulsive as it changes from concave to relatively flat upon maltose binding (Figure 2B). As such, there will likely be less inter-domain steric pull on the TEM-1 β3 fusion section by MBP when maltose is bound.

### Insights into the (Maltose Absent) apo RG13 Structure

There are three points of evidence that indicate that the N-terminal half of the β3 strand of TEM-1 in RG13 in the absence of maltose is in a more tensed, non-wt position. Firstly, this stretch of residues could not be assigned to the wt TEM-1 position using NMR measurements on RG13 in the absence of maltose but, upon maltose binding, changes conformation and could be assigned [8]. Second, the ease of dislodging the β3 strand, and adjacent β4 strand region, by binding zinc at mM concentrations at an interdomain site as evidenced in the RG13 crystal structures suggests that some strain or partial displacement is already present in this β3 region. Thirdly, the molecular modeling of an intact TEM-1 within the RG13 framework indicated a very close proximity between the TEM-1 and MBP domains since the fusion points on either domain are not on protruding, flexible loop regions. We therefore hypothesize that, in the absence of maltose and zinc, that by MBP sterically pulling slightly on the TEM-1 β3-strand, which starts at TEM-1 residue F230, this strand is modestly shifted in maltose-free RG13 affecting both *k*_cat_ (oxyanion needed) and *K*_m_ (carboxyl moiety interaction needed). Since the N-terminal part of the β3 strand is very close to both K234 and A237 of TEM-1 located in the middle of the β3 strand (even though these two residues were assigned in the NMR spectra), subtle changes in their dynamics and position could account for the 25-fold maltose-dependent activity switching of RG13. A slight pulling on the N-terminal end of the β3 by a maltose-free MBP domain in RG13 could affect the position of the important oxyanion hole, involving A237, by perhaps 0.1–0.4Å which will both have an effect on *K*_m_ and *k_cat_* (as the substrate needs to place its carbonyl oxygen in this site and also it needs to be primed for ∼2 steps during catalysis (each only 5-fold so changes need to be small). Shifts of that magnitude in the oxy-anion hole residues of a closely related β-lactamase [17] have been observed to affect *k*_cat_ in the 5-fold range [18].

We note that TEM-1 residue E166 was previously suggested to be functionally perturbed prior to maltose binding due to (a) a decreased deacylation of RG13 in the absence of maltose and (b) disappearance of the E166 peak in the NMR spectra of apo-RG13 (although this peak in the HSQC-TROSY of RG13 in the presence of maltose was however already quite weak) [7], [8]. Residue E166 has a key role in deacylation [19] and therefore seem to be a candidate residue to be involved in maltose switching in RG13 yet the structure found this residue to be ordered and quite distant from the fusion site. We do however not rule out an indirect effect on the conformation of E166 via β3 strand changes as this strand interacts with the E166-containing Ω loop. Such β3-strand mediated Ω-loop changes were speculated for the G238S mutant of TEM-1 [20] and we also recently observed a large Ω loop displacement in variants of a closely related β-lactamase [21] (although such a large loop shift was not observed in RG13 by NMR [8]).

### MBP Fusion Site

Our mutagenesis studies indicate that proper anchoring of the linkers to MBP, via R316, S585 and even A586, is critical to transmit the maltose signal as altering these residues led to loss of maltose-switching (Figure 4). The mutations involving these residues likely caused these anchor regions to be no longer tightly interacting with MBP such that MBP no longer can control the activity of TEM-1 thereby explaining their abrogated maltose switching. This maltose switching is likely done by a positional change, as illustrated by the shift in anchor points (Figure 5A), and/or by relieving steric clashes between the concave surface of the MBP domain upon maltose binding (Figure 2B). Previously, the MBP mutation A319T (RG13 residue A586) was also found to cause a loss of maltose switching [7]. This mutation is adjacent to the S585 linker anchor residue and, since there is no space to accommodate the threonine, this mutation will likely lead to a shift of these few residues thereby likely displacing S585 from its anchoring role. In addition to stable anchor residues, linker length was also critical for switching as introducing slack into linker 1 via a glycine insertion led to loss of switching as the maltose-free activity increased to wt TEM-1 activity. Furthermore, by decreasing slack in the linker 2 region, via deletion of G584, the maltose-free activity could be further dampened and maltose switching increased. Proper linker anchoring and linker length are thus critical attributes for maltose switching by RG13.

In addition to RG13, another TEM-1 MBP fusion protein has previously been obtained that uses the same MBP fusion site of residue 316 to also obtain maltose-dependent signaling [2]. But instead of TEM-1 residue 229 being fused to MBP residue 316, this other MBP-TEM-1 construct fused TEM-1 residue N170 to MBP residue 316. This suggests that this MBP region, near residue 316, has unique and robust features that can transmit maltose-dependent conformational changes to an unrelated protein in different ways. This other MBP-TEM-1 maltose-regulated fusion construct was shown to have its maltose-dependent abilities to be negatively affected by increasing the linker length by one and two additional residues [2]. This agrees with our RG13 structure-based allostery hypothesis as the linker length and amount of slack are hypothesized to be key for proper maltose signaling in RG13. Another example indicating that short linkers tend to have better switching properties in engineered allosteric fusion proteins is a TEM-1:cytochrome *b*_562_ construct [22], [23].

**Figure 7:**
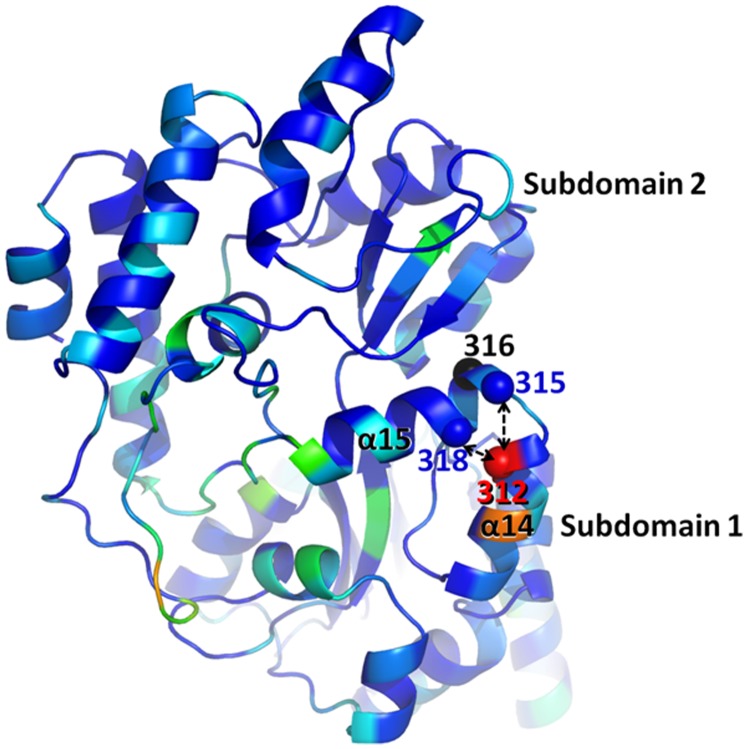
Structure of uncomplexed MBP (1OMP) showing maltose-induced local Cα-Cα distance shifts. Maltose-dependent Cα-Cα distance differences for residues i = i+3 and i = i+6 are averaged and mapped onto the structure with residues color ramped from blue to red for averaged shifts ranging from 0 to 1.4Å, respectively. Residue 312 yielded the largest maltose-dependent local shifts (1.4Å). Labeled also are residues 315 (i+3) and 318 (i+6) with arrows to residue 312 (i) indicating the local distances that were maximally shifted by maltose. Helices α14 and α15 are labeled as well as residue 316 (black), the site of the RG13’s TEM-1 insertion in MBP, to show that the insertion occurred within the maltose-dependent locally shifting region of 312–318 situated between the two helices. Labeled residues are also shown as spheres. The orientation of MBP is similar to that of Figure 5.

It is interesting as to why MBP residue 316 is selected for during the generation of two different maltose-regulated TEM-1 fusion constructs as residue 316 is not near where the largest conformational differences are observed upon maltose binding that include a 35° domain closure conformational change. Residue 316 is actually situated on the backside of the hinge region of the bi-lobal MBP structure. However, one has to consider as to how these allosteric fusion proteins are generated as the TEM-1 domain is inserted into a single site of MBP (with possibly some residues deleted as in RG13, Figure 1). Although the linker anchor points of MBP are not where there are the largest differences in distance between an uncomplexed and complexed MBP are found, the anchor points are near where the largest differences are when one only considers Cα positions of a few residues downstream from each other in the continuous polypeptide chain to allow for insertion of the non-homologous TEM-1 gene at a single site. By calculating Cα-Cα Δdistance measurement of residues i and i+x via substracting the distances from the maltose-bound from the maltose-free MBP structure, the MBP region at residue 312 lights up as to having the largest Cα-Cα difference for i and i+3 and i and i+6 (Figure 7). These calculations suggest that the MBP region from 312 to 312+i (i.e. 312–318) has the largest shifts thereby likely explaining why insertions near residue 316, in particular since R316 has adopted the linker anchor role in RG13 (Figure 2C), are capable of transmitting maltose signals to TEM-1 (as is also evident mechanistically in Figure 5A). An additional potential benefit of this region, being on the backside of MBP, is that this region could exert a lever/torque effect as the fusion points are close to the hinge region. As such, this region is thus perhaps able to exert a larger force on the movements near its rotation point, which might be needed as the β3 TEM-1 strand is normally well lodged into the active site. Furthermore, the backside of MBP, near the hinge region where TEM-1 is attached, changes from concave to more flat upon maltose binding (Figure 2B) and could therefore decrease steric contacts between TEM-1 and MBP and thus provide additional slack for the β3 linker regions such that they can adopt the *wt* catalytically competent conformation. It is remarkable that the forced evolutionary pressure resulted in this successful RG13 of having indeed found the MBP 312–318 region and the 230 β3-strand region of TEM-1 indicating that this approach works to find this narrow signaling window region being almost a ‘needle in a hay stack’.

### Zn^2+^ Binding and Regulation in RG13

Biochemical and mutagenesis studies indicated that Zn^2+^ inhibits RG13 in a non-competitive, yet reversible manner and suggested the presence of a single low µM Zn^2+^ site with an inhibitory role for residues H382 (*H26*) and H375 (*H289*) [6]. These residues likely have an indirect role as mutating these residues did not affect Zn^2+^ binding, only Zn^2+^ inhibition. In agreement, the structure of RG13 revealed also no direct involvement of these histidine residues in Zn^2+^ binding even with mM Zn^2+^ concentration. Instead, the RG13 structure, with 2.5 mM Zn^2+^ present, revealed a Zn^2+^ binding role and potentially regulatory role for role for residues D164, H468 and potentially E477. However, mutating 2 or 3 of these residues only caused a minor effect (2- to 4-fold; Table 2) on the *K_i_* for Zn^2+^ indicating no exclusive role for D164/H468/E477 in the Zn^2+^ inhibition mechanism. In agreement, previous mutagenesis studies found that the H452A/H468L mutation pair did not alter Zn^2+^’s inhibitory effect [6]. The observed second Zn^2+^ site, which also involves residues from two neighboring crystallographically related RG13 molecules (in the P1 space group), is likely also not functionally relevant for RG13 as mutating H509 (*H153*) or H514 (*H158*) did not affect Zn^2+^ inhibition [6].

There are three possible explanations for the apparent discrepancy between the mutagenesis results and the crystallographically observed Zn^2+^ binding site in RG13. First, Zn^2+^ binding to RG13 could be kinetically controlled such that at early time points, the lowest energy barrier binding site will be occupied first whereas the energetically most favorable binding site might take longer to reach due to a higher initial energy barrier. The latter state could perhaps be reached during the several days it took for RG13 crystals to form. This could be compounded by the difference in “induced-fit” and “conformational selection” as modes of binding [24]. At low concentrations, conformational selection takes place, whereas at higher concentrations, induced fit takes place (i.e. ligand binding first to low affinity conformation thereby inducing conformational change). A second possibility is that only the observed zinc-bound conformations could be crystallized such that crystallization selected for a particular conformation that might perhaps not be the dominant one in solution. A third possible explanation is that there is more than one low µM inhibitory zinc binding site that are all mutually exclusive since they involve juxtapositioning of 1–2 liganding residues of MBP and 1–2 liganding residues of TEM-1. This last possibility is especially intriguing as previous mutagenesis experiments, indirectly, indicated that the presumed single Zn^2+^ binding site in RG13 would fall into a rare category of Zn^2+^ binding sites: single and double mutant studies had ruled out all histidine and cysteine residues in RG13 being involved in zinc binding [6] if the hypothesis were correct regarding a single unique inhibitory Zn^2+^ site. However, a Zn^2+^ binding site without histidine and without cysteine is quite rare as it has only been observed in ∼1% out of the over 230 different Zn^2+^ sites [25]. Thus, if there is indeed a single unique non-histidine/non-cysteine Zn^2+^ inhibitory site in RG13, it would fall into this rare category that could not even be observed crystallographically at mM zinc concentrations in the RG13 structures whereas two different, histidine-containing, Zn^2+^ sites were observed instead. Therefore, an alternative interpretation is that RG13 has multiple, yet mutually exclusive low µM inhibitory zinc binding sites that likely will have one or more histidine liganding residues (cysteine residues are occupied in disulfide bonds).

That there are multiple mutually exclusive µM Zn^2+^ inhibitory sites is an attractive hypothesis for two additional reasons. First, RG13 has a potential mechanism to form multiple mutually exclusive inhibitory Zn^2+^ binding sites via mere rotation and repositioning of the MBP and TEM-1 domains to juxtaposition 1–2 Zn^2+^ liganding residues from each domain. Such juxtapositioning could perhaps entail Zn^2+^ first binding to one of the (histidine-containing) multi-residue “half-sites” situated on one domain (i.e. H468/E477, H509/H514, or Y17/H39; see Figure S2) followed by 1–2 additional ligands from the other domain such as D164 combining with H468/E477 (Figure S2). Note that µM Zn binding can be readily be engineered into a variety of proteins via juxtapositioning of at least 2 His residues [26]–[29]. Due to avidity, all RG13 would need to do is to rotate, and perhaps shift the two domains somewhat such that His/Asp/Glu and/or other Zn^2+^ liganding residues from the different domains are juxtaposed locking the RG13 in a more fixed conformation thus affecting the critical linker regions. As RG13 has a low pI of 5.53, it has ample opportunity to do this via 40 Asp, 47 Glu, and 9 His residues such that 15% of its residues can be involved in liganding Zn^2+^ (96 out of 637 residues). Since most of these residues are at the surface, the relative amount of potential Zn^2+^ liganding residues is thus even higher. We hypothesize that the multiple yet mutually exclusive inhibitory Zn^2+^ sites of RG13 share common features such as bridging both TEM-1 and MBP and also affecting the critical inter-domain linker regions. A second additional reason for the multiple mutually exclusive Zn^2+^ sites hypothesis is that it provides an explanation for the mutagenesis results as the double and triple mutant neither fully nor negligibly affected the *K_i_* (2- to 4-fold change was observed); these mutants likely thus eliminated one of the inhibitory Zn^2+^ sites from the population causing a partial Zn^2+^ binding site redistribution. Note that inter-domain Zn^2+^ binding is the most likely possibility as MBP and TEM-1 themselves as individual proteins are not regulated by Zn^2+^ [6]. Also, if the µM Zn^2+^ binding site were located within just one of the domains, some Zn^2+^ electron density would have likely become apparent due to the mM Zn^2+^ concentration used during crystallization. It is also not uncommon to have two mutually exclusive zinc binding sites as this was observed previously in an engineered zinc biosensor [30]. This third possibility to explain the discrepancy between the mutagenesis data and the RG13 structure would thus render the RG13:Zn^2+^ crystal structure as one of the two or more modes of Zn^2+^ inhibition in RG13 with individual Zn^2+^ affinities such that the apparent *K_i_* shifts by about 3-fold upon mutating the crystallographically observed one (from 2µM to 4–8µM; Table 2). Future experiments are needed to further probe these three possible explanations.

Regarding the indirect role of H382 (*H26*) and H375 (*H289*) in Zn^2+^ inhibition [6], we hypothesize that the above noted close steric proximity between the MBP and TEM-1 domains could explain why mutating TEM-1 residues H26 and/or H289 had an effect on Zn^2+^ inhibition but not on Zn^2+^ binding [6]. Each of these histidines is at the end of a helix either after or before the GSGGG loop. Their side chains both protrude to the solvent forming an aromatic stacking interaction with each other (Figure 6). We postulate that mutating either or both of these histidines to alanine results not only in removal of a sterically protruding histidine sidechain, but could also lead to a shift of the other histidine concomitant with shift or disorder of this entire engineered loop region comprising residues **H_375_**W_376_GSGGG**H_382_ (**H375 is TEM-1 residue *H289* and H382 is TEM-1 residue *H26*)_._ This loop region is adjacent to the fusion site (Figure 6) and being potentially less protruding and less ordered upon a H→A mutation could lessen the steric repulsion between the MBP and TEM-1 domains in that region and thus allow Zn^2+^ to bind to the interdomain site(s) without having to dislodge the β3-β4 region when the MBP and TEM-1 domains pivot to juxtaposition Zn^2+^ liganding residues. An alternative explanation, but sharing similar features, is that the histidine mutation(s) cause destabilization of the circularly-permutated loop region that could alter the W376(*W290*)-W317(*W229*) interaction (Figure 6B). These tryptophan residues in TEM-1 are not tolerant to substitution [13]; W229 is near the fusion site (Figure 1) and is displaced 25Å in RG13 compared to its position in the *wt* TEM-1 structure. A H26A and/or H289A mutation could thus indirectly disrupt the W229:W290 interaction thereby affecting whether the critical β3-β4 region will be dislodged when inter-domain Zn^2+^ is bound.

Here we propose a mechanistic model for RG13 from the above findings and discussion: The N-terminal section of the β3 strand adjacent to the TEM-1 228 fusion point is likely strained or partially displaced in RG13. This strain in the absence of maltose is likely due to a combination of the close proximity of the TEM-1 and MBP domains when fused in RG13 that is exacerbated by the concave character of that MBP domain surface and the increased distance between the anchor points. This tensed state of the TEM-1 domain can undergo two different allosteric pathways. First, activation via relieving the strain by maltose binding will alleviate the steric repulsion between the MBP and TEM-1 domains due to flattening the surface near the MBP fusion site from its concave state in the absence of maltose. Furthermore, maltose binding will change the position of MBP helices α14 and α15 such as to shorten the distance between the anchor points about 2Å. A second pathway the strained apo-RG13 molecule could undertake is inhibition via Zn^2+^ mediated reorientation of the MBP and TEM-1 domains by forming a 3^rd^ (Zn^2+^-mediated) contact point between these domains (in addition to the two linkers already present in RG13). This would allow the strained β3 strand region to completely pop out of its strained wt-type-like position as the inter-domain bridging zinc ion ‘twist-ties’ the linkers to gain the needed additional linker slack for Zn^2+^ to bind to both domains (at multiple yet mutually exclusive sites). The described findings regarding maltose and Zn^2+^ regulation in an engineered fusion protein could be useful for future allosteric protein engineering efforts.

## Materials and Methods

### Expression and Purification

RG13 was expressed and purified as previously described [6]. Briefly, the expression plasmid containing RG13 was transformed into *Escherichia coli* strain PM9F’. The culture were inducted by 1 mM IPTG and grew overnight at 20°C. Cells were lysed by French press and RG13 was subsequently purified using an amylase affinity resin (New England Biolabs) and eluted using maltose. The eluted RG13 protein was dialyzed at 4°C, to remove maltose, and subsequently concentrated to ∼11 mg/mL in 20 mM Tris-Cl pH7.0 before mixing with glycerol for storage at −80°C. Aliquots of RG13 were thawed and subjected to gel filtration using a Superdex200 column (GE Biosciences) that was equilibrated with 20 mM Tris/HCl pH 7.2 and 500 mM NaCl. RG13 was subsequently buffer exchanged to 10 mM Tris/HCl pH 7.2, and concentrated to 10–15 mg/ml, as assessed by the Bradford assay, prior to crystallization. RG13 mutants were expressed as above with the following exceptions. The strain used was BL21, the overnight growth temperature was 25°C, and purification was performed using an MBPTrap column (GE Biosciences).

### Crystallization and Soaking

Prior to crystallization, ZnCl_2_ was added to RG13 to a final concentration of 2.5 mM. Initial crystallization conditions were found by using Hampton screen kits. After optimization, single crystals were obtained by vapor diffusion sitting drop method at room temperature: a 1.0 µl drop was prepared using 0.5 µl protein mixture (13.8 mg/ml RG13 and 2.5 mM ZnCl_2_) and 0.5 µl reservoir solution (containing 0.2M Ammonium Acetate, 0.1M Tris pH 8.5–9.5, 15–30% PEG3350) and equilibrated over a 1 ml reservoir solution. RG13-Zn co-crystals grew to full size in one week and were subsequently cryo-protected in the reservoir solution with 33%PEG3350 and 2.5 mM ZnCl_2_.

### Data Collection and Structure Determination

Crystals of RG13 grew in two different space groups. Diffraction data for the RG13-Zn complex P1 and C2 spacegroup crystals were collected at the National Synchrotron Light Source X-29A and processed using HKL2000 [31], [32]. The data from the P1 space group was double checked but could not be processed in a higher symmetry space group. The structures for each of the space groups was solved by Molecular Replacement using the program Phaser [33] with two RG13 molecules in the P1 space group and one RG13 molecule in C2 space group. Based on the folding pattern of MBP of having two subdomains and the circular permutation of RG13 [5], molecular replacement searches were carried out using search models encompassing each of the MBP subdomains and most of the TEM-1 structure to search for the two copies of RG13 molecules in the asymmetric unit of P1 space group. The search models were obtained from the MBP structure (PDBid 1OMP) and the TEM-1 structure (PDBid 1ZG4). The Molecular Replacement program Phaser [33] successfully placed the subdomains in the asymmetric unit of P1 and C2 space groups to yield two and one RG13 molecules, respectively, in the asymmetric unit. After several rounds of REFMAC (CCP4 suite [34]) refinement and adjusted using COOT [10], most residues of the full length RG13 protein in both space groups could be built. During the refinement, inspection of Fourier difference maps indicated the presence of very strong non-protein electron density peaks which were identified, and refined subsequently, as zinc ions. Water molecules were included in the final stages of refinement. The simulated annealing protocol in CNS [35] was also used in refinement. Iterative rebuilding and refinement allowed the final model to converge with an R/R_free_ 23.2/29.2% for the P1 space group and 23.3/29.1% for the C2 space group (Table 1 for more refinement stats). The C2 space group contains RG13 residues 1–316, 328–582, 585–637, 2 zinc ions, and 134 water molecules whereas the P1 space group structure contains two RG13 copies of residues 1–637, 4 zinc ions, and 490 water molecules. PROCHECK [36] and DDQ [37] were used during the model building process and to validate the final structures. The atomic coordinates and structure factors of the RG13 with zinc ions for the P1 and C2 space groups have been deposited in the Protein Data Bank under accession code 4DXB and 4DXC, respectively. For the rigid body positioning of an intact TEM-1 domain within a RG13 framework, we used the program COOT and the coordinate files as described in the results section.

### Biochemical Characterization of RG13 and RG13 Mutants

The *K*_i_ for Zn^2+^ for was determined as previously described by nitrocefin hydrolysis assay [6]. Maltose-induced switching was determined at 25°C by nitrocefin hydrolysis assay with and without maltose in which the initial rate of nitrocefin hydrolysis was monitored at 486 nm as previously described [5]. Solution conditions were 100 mM sodium phosphate buffer (pH 7.4) with 26 nM protein, 100 µM nitrocefin, and 0 or 10 mM maltose.

## Supporting Information

Figure S1 Electron density for active site and linker regions of RG13 (A) 2*F*_o_-*F*_c_ density map depicting the TEM-1 active site region; (B) 2*F*_o_-*F*_c_ density maps for residues 315–330 (the linker 1 region) and (C) residues 571–587 (the linker 2 region); (D) 2*F*_o_-*F*_c_ density map for residues 377–381 (the engineered GSGGG linker); (E) Temperature representation of the RG13 structure in the P1 space group. The thicker and the more red the backbone is, the more flexible it is due to its higher refined temperature factors. All density maps shown are contoured at 1.0σ.
(TIF)

Figure S2 Zinc ion binding sites in RG13. (A) main zinc ion RG13 binding site in space group C2 structure bridges MPB domain and TEM domains of RG13 involving residues D164, H468, and E477. (B) The main zinc ion binding site in space group P1 involves residues D164 and H468; (C) Second zinc ion binding site in the C2 space group structure involving residues of H509 and H514 in the TEM domain of RG13. (D) The second zinc binding site in the P1 space group involving H509, H514, Y17′, and H39′ (the latter two belong to a crystallographically related RG13-MBP domain). The 2*F*_o_-*F*_c_ density maps were contoured at 1.5σ and colored blue for the amino acid residues of RG13 and the water molecules. Omit *F*_o_-*F*_c_ maps were contoured at 10σ and colored green for the Zn ions. Domain coloring is same as in Figure 1 with the crystallographic related neighboring molecules depicted in grey. Water molecules and zinc ions are shown as red and grey spheres, respectively.
(TIF)
