# Supplementary figures and images for: Structure of an Engineered β-Lactamase Maltose Binding Protein Fusion Protein: Insights into Heterotropic Allosteric Regulation
Source: PLoS One. 2012 Jun 14;7(6):e39168. doi: 10.1371/journal.pone.0039168 (PMC3375305; doi:10.1371/journal.pone.0039168)

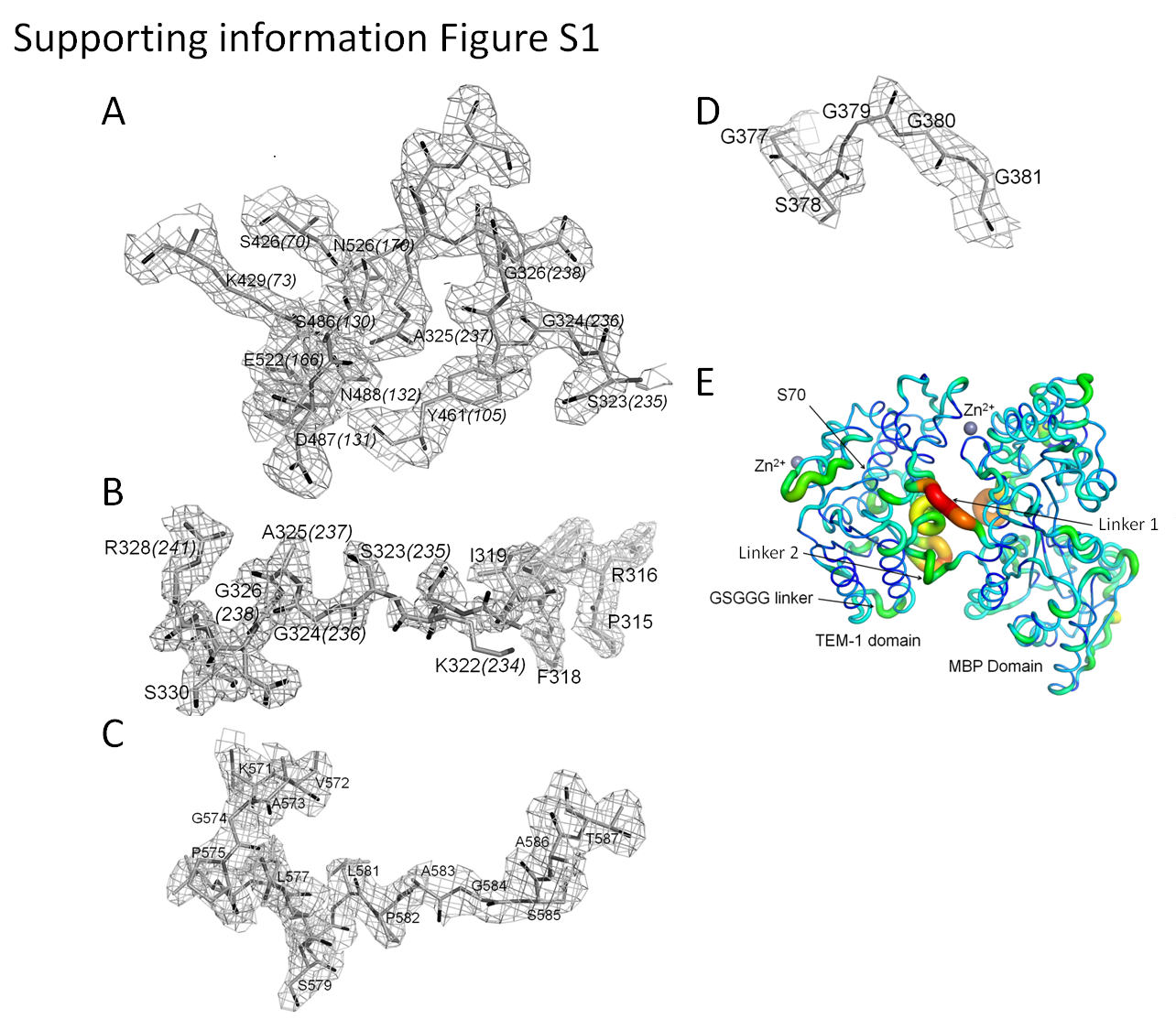

Supplement: Figure S1 — Electron density for active site and linker regions of RG13 (A) 2F o-F c density map depicting the TEM-1 active site region; (B) 2F o-F c density maps for residues 315–330 (the linker 1 region) and (C) residues 571–587 (the linker 2 region); (D) 2F o-F c density map for residues 377–381 (the engineered GSGGG linker); (E) Temperature representation of the RG13 structure in the P1 space group. The thicker and the more red the backbone is, the more flexible it is due to its higher refined temperature factors. All density maps shown are contoured at 1.0σ. (TIF) [file pone.0039168.s001.tif]

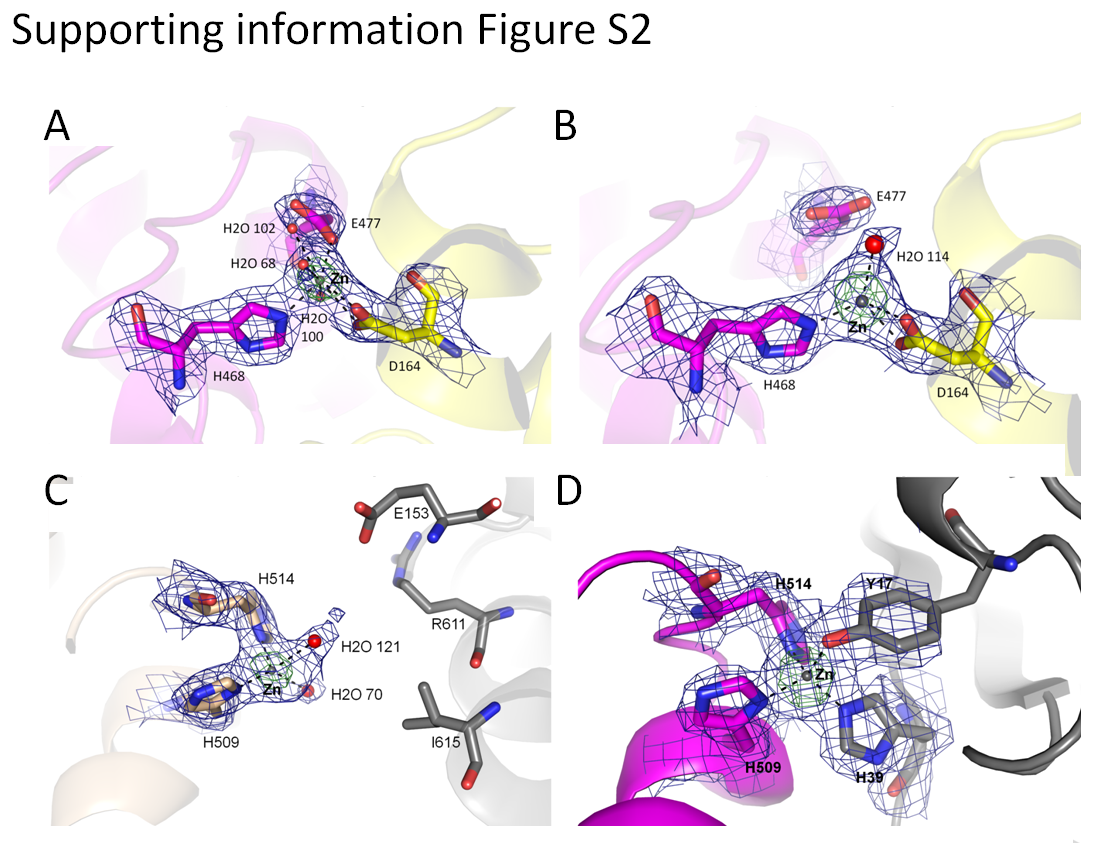

Supplement: Figure S2 — Zinc ion binding sites in RG13. (A) main zinc ion RG13 binding site in space group C2 structure bridges MPB domain and TEM domains of RG13 involving residues D164, H468, and E477. (B) The main zinc ion binding site in space group P1 involves residues D164 and H468; (C) Second zinc ion binding site in the C2 space group structure involving residues of H509 and H514 in the TEM domain of RG13. (D) The second zinc binding site in the P1 space group involving H509, H514, Y17′, and H39′ (the latter two belong to a crystallographically related RG13-MBP domain). The 2F o-F c density maps were contoured at 1.5σ and colored blue for the amino acid residues of RG13 and the water molecules. Omit F o-F c maps were contoured at 10σ and colored green for the Zn ions. Domain coloring is same as in Figure 1 with the crystallographic related neighboring molecules depicted in grey. Water molecules and zinc ions are shown as red and grey spheres, respectively. (TIF) [file pone.0039168.s002.tif]
